# Oral histoplasmosis

**DOI:** 10.5935/0103-507X.20170057

**Published:** 2017

**Authors:** Gustavo Antonio Correa Momesso, Tárik Ocon Braga Polo, Valthierre Nunes de Lima, Cecília Alves de Sousa, Ana Maria Pires Soubhia, Ellen Gaetti Jardim, Leonardo Perez Faverani

**Affiliations:** Division of Oral and Maxillofacial Surgery, Department of Surgery and Integrated Clinic, Faculdade de Odontologia de Araçatuba, Universidade Estadual Paulista “Julio de Mesquita Filho” - Araçatuba (SP), Brazil.; Division of Oral and Maxillofacial Surgery, Department of Surgery and Integrated Clinic, Faculdade de Odontologia de Araçatuba, Universidade Estadual Paulista “Julio de Mesquita Filho” - Araçatuba (SP), Brazil.; Division of Oral and Maxillofacial Surgery, Department of Surgery and Integrated Clinic, Faculdade de Odontologia de Araçatuba, Universidade Estadual Paulista “Julio de Mesquita Filho” - Araçatuba (SP), Brazil.; Division of Oral and Maxillofacial Surgery, Department of Surgery and Integrated Clinic, Faculdade de Odontologia de Araçatuba, Universidade Estadual Paulista “Julio de Mesquita Filho” - Araçatuba (SP), Brazil.; Division of Oral Patology, Faculdade de Odontologia de Araçatuba, Universidade Estadual Paulista “Julio de Mesquita Filho” - Araçatuba (SP), Brazil.; Faculdade de Odontologia, Universidade Federal de Mato Grosso do Sul - Campo Grande (MS), Brazil.; Division of Oral and Maxillofacial Surgery, Department of Surgery and Integrated Clinic, Faculdade de Odontologia de Araçatuba, Universidade Estadual Paulista “Julio de Mesquita Filho” - Araçatuba (SP), Brazil.

To the editor,

Histoplasmosis is an opportunistic fungal infection, endemic to Latin America, that is caused by *Histoplasma capsulatum*. This infection mostly occurs in the lungs^(1)^ and is frequently associated with AIDS.^(2)^ Sometimes, histoplasmosis is misinterpreted with tuberculosis due to their clinical similarities.^(3,4)^ Oral involvement is very rare in histoplasmosis, being associated only with its disseminated form.^(5)^

**Figure 1:**
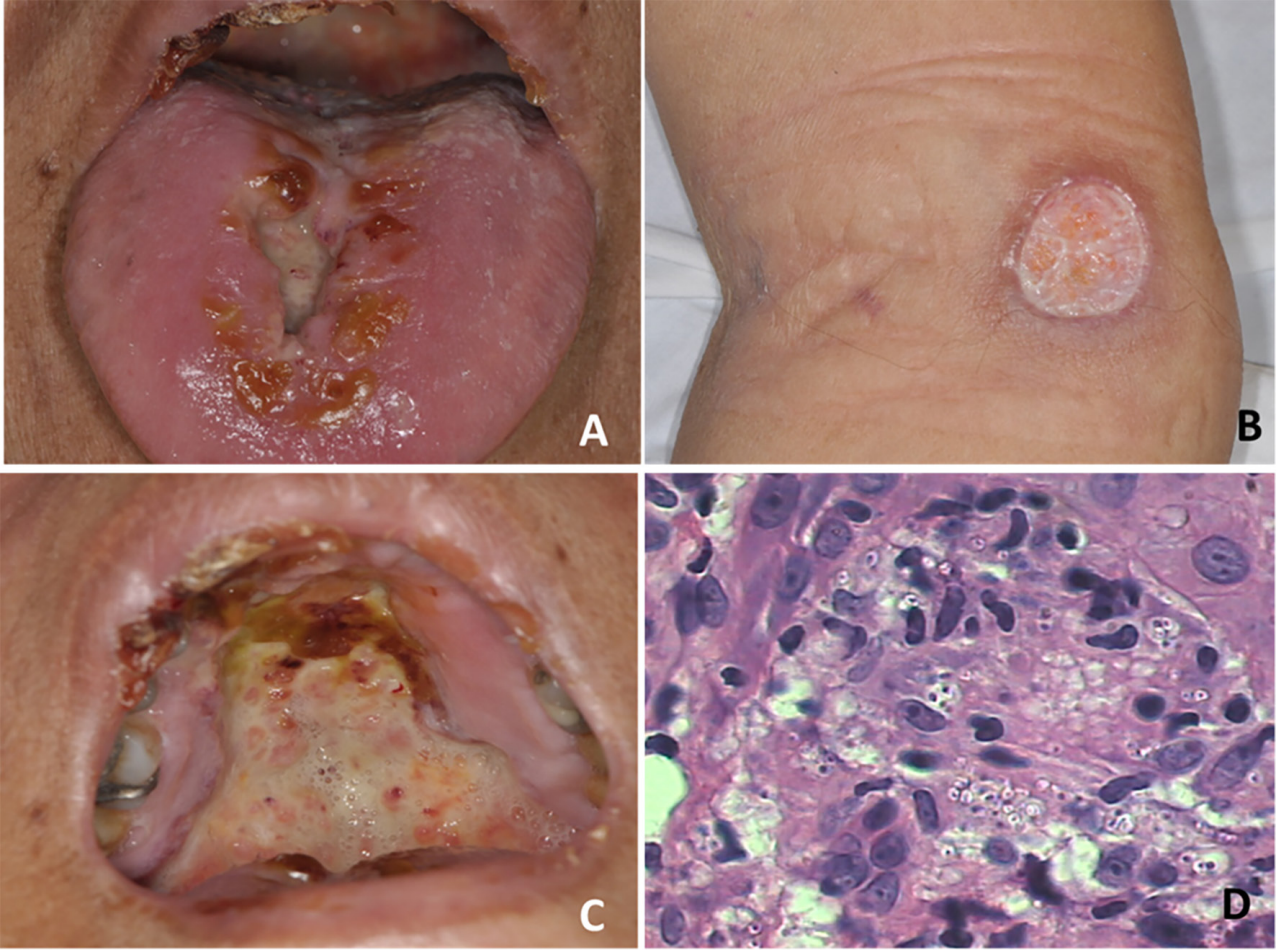
(A to C) Lesions scattered on the tongue, hard palate and skin. (D) Histopathological analysis showed the presence of clear haloes and hyphae.

A 60-year-old white patient was referred to the Oral and Maxillofacial Surgery team of the *Faculdade de Odontologia* of the *Universidade Estadual Paulista "Julio de Mesquita Filho"* (UNESP, Araçatuba, SP, Brazil) for evaluation of lesions scattered on the tongue, hard palate and skin. Clinical examination showed a prostrated and immunosuppressed patient with feeding difficulty. Oral examination revealed ulcerated lesions, caries and purulent exudate on the dorsal surface of tongue and on the hard palate. In addition, an erosive whitish lesion, typical of opportunistic lesions, was observed on the skin of the left forearm. A tongue biopsy was carried out for histopathological analysis in addition to the routine laboratory exams that were prescribed. The rapid HIV test and the specific test (Western blot) were positive, confirming a diagnosis of AIDS (Figures 1A, 1B and 1C).

The routine histopathological analysis showed the presence of clear haloes and hyphae, which, in combination with the clinical characteristics, confirmed a diagnosis of histoplasmosis (Figure 1D). Drug treatment was initiated with three daily mouth washes of nystatin (500,000IU), and the patient was referred to the medical clinic for AIDS treatment. A month later, the family reported that the patient had died.

Early diagnosis of histoplasmosis is important for improving the patient's quality of life. The timely discovery of oral lesions helps physicians treat the symptoms of HIV-positive patients, in addition to being a clinical predictor of AIDS with systemic symptoms. In the present case, the patient's delayed search for medical assistance led to a late diagnosis, which was decisive for the case prognosis.

Gustavo Antonio Correa Momesso *Division of Oral and Maxillofacial Surgery, Department of Surgery and
Integrated Clinic, Faculdade de Odontologia de Araçatuba, Universidade
Estadual Paulista "Julio de Mesquita Filho" - Araçatuba (SP),
Brazil.* Tárik Ocon Braga Polo *Division of Oral and Maxillofacial Surgery, Department of Surgery and
Integrated Clinic, Faculdade de Odontologia de Araçatuba, Universidade
Estadual Paulista "Julio de Mesquita Filho" - Araçatuba (SP),
Brazil.* Valthierre Nunes de Lima *Division of Oral and Maxillofacial Surgery, Department of Surgery and
Integrated Clinic, Faculdade de Odontologia de Araçatuba, Universidade
Estadual Paulista "Julio de Mesquita Filho" - Araçatuba (SP),
Brazil.* Cecília Alves de Sousa *Division of Oral and Maxillofacial Surgery, Department of Surgery and
Integrated Clinic, Faculdade de Odontologia de Araçatuba, Universidade
Estadual Paulista "Julio de Mesquita Filho" - Araçatuba (SP),
Brazil.* Ana Maria Pires Soubhia *Division of Oral Patology, Faculdade de Odontologia de Araçatuba,
Universidade Estadual Paulista "Julio de Mesquita Filho" - Araçatuba
(SP), Brazil.* Ellen Gaetti Jardim *Faculdade de Odontologia, Universidade Federal de Mato Grosso do Sul - Campo
Grande (MS), Brazil.* Leonardo Perez Faverani *Division of Oral and Maxillofacial Surgery, Department of Surgery and
Integrated Clinic, Faculdade de Odontologia de Araçatuba, Universidade
Estadual Paulista "Julio de Mesquita Filho" - Araçatuba (SP),
Brazil.*
